# From Microbial Ecology to Functional Components in Microbe–Host Interactions

**DOI:** 10.3390/biology15080635

**Published:** 2026-04-17

**Authors:** Tao Wang, Zhengjin Wang, Xiao Yang, Lei Zhang

**Affiliations:** 1 Microbiome-X, School of Public Health, Cheeloo College of Medicine, Shandong University, Jinan 250012, China; wtwangtao163@163.com (T.W.); wangzz0537@163.com (Z.W.); yangxiao6@mail.sdu.edu.cn (X.Y.); 2 School of Pharmaceutical Engineering, Jining Medical University, Jining 272067, China

**Keywords:** microbial functional components, microbe–host interactions, clinical translation, biomarkers

## Abstract

Microbiome research is shifting from a focus on “whole microorganisms” to an emphasis on microbial functional components. This review systematically describes how the effects of microbial communities on the host are mediated by bioactive functional components released by microbes. These components primarily exert their effects through interactions with host Pattern Recognition Receptors (PRRs) and metabolic sensing receptors, thereby regulating host immune, metabolic, and barrier function networks. The biological effects of these functional components are highly context-dependent. Under homeostasis, metabolites such as SCFAs and bile acids promote mucosal immune tolerance and maintain epithelial barrier integrity. However, the same signals can become deleterious under dysbiosis, driving inflammation and contributing to colorectal tumorigenesis. Mechanistic dissection of individual components, such as lipopolysaccharide (LPS), is now propelling a transition in clinical translation from whole-microbe-based interventions toward component-oriented diagnostics and therapeutics. Component-oriented diagnostics and therapeutics use defined microbial molecules rather than whole microorganisms. Microbial nucleic acids (e.g., HPV DNA), metabolites (e.g., SCFAs), and proteins can serve as biomarkers for disease risk, diagnosis, and prognosis. Therapeutic strategies include targeted modulation of beneficial components, neutralization of harmful molecules, and engineered microbial delivery.

## Simple Summary

Microbiome research has often focused on identifying which microorganisms are present in the body. However, microbes influence health not only as whole organisms but also through the bioactive functional components they produce. These functional components, including nucleic acids, metabolites, proteins, cell wall structures, and extracellular vesicles, directly interact with host immune and metabolic systems. For example, short-chain fatty acids (SCFAs), a class of microbial metabolites, can modulate host immune responses by promoting regulatory T cell differentiation. In this review, we summarize current understanding of how the host senses and integrates these microbial-derived signals in a context-dependent manner, shaping outcomes ranging from homeostasis to disease. Emphasizing functional components rather than microbial identity may provide clearer mechanistic insight and support the development of more precise diagnostic and therapeutic strategies, including nucleic acid-based biomarkers for pathogen detection such as HPV testing and engineered microbial systems for targeted therapeutic delivery.

## 1. Introduction

A substantial body of evidence has established a close association between microbial communities and host health and diseases [1]. Traditionally, research in this field has centered on the concept of the “whole microorganism.” This perspective is reflected both in the organism-based definition of pathogens rooted in Koch’s postulates and in contemporary intervention strategies that rely on intact microbial consortia, such as probiotics and fecal microbiota transplantation (FMT) [2,3]. However, approaches that treat microorganisms as discrete, organism-level units face inherent limitations when attempting to delineate specific mechanisms of action. For example, a single microorganism may produce multiple bioactive functional components with distinct or even opposing effects, making it difficult to attribute host responses to the organism as a whole [4,5]. Moreover, many host-modulating effects are mediated by specific microbial molecules, such as metabolites or structural components, which cannot be resolved at the organism level [6,7]. As a result, changes in community composition at the population level are often difficult to map precisely onto defined host physiological or pathological phenotypes [8,9,10]. These limitations are also reflected in clinical applications. Interventions based on live microorganisms, including probiotics and FMT, frequently show variable therapeutic efficacy, largely due to inter-individual differences in baseline microbiota and the complexity of microbe–host interactions [3,11,12]. Furthermore, the precise molecular mechanisms underlying these interventions remain poorly defined [13].

In recent years, increasing mechanistic insights have suggested that the impact of microorganisms on the host is mediated not solely by the presence of intact microbes, but largely through specific bioactive molecules that they synthesize, modify, and release, hereafter referred to as functional components [14]. These functional components encompass microbe-associated molecular patterns (MAMPs), such as lipopolysaccharide (LPS) and peptidoglycan; metabolites, including SCFAs and bile acids; as well as microbial proteins, nucleic acids, and outer membrane vesicles (OMVs) [15,16,17]. Functionally, these components engage host sensing and signaling systems: MAMPs act as ligands for Pattern Recognition Receptors (PRRs) such as Toll-like receptor 4 (TLR4) and nucleotide-binding oligomerization domain-containing protein 2 (NOD2) [18,19]; metabolites regulate immunometabolic homeostasis through receptors such as G protein-coupled receptors (GPCRs), including GPR41 and GPR43 [20,21,22], and vesicles facilitate the delivery of diverse microbial cargo [23]. Through these interactions, microbial functional components modulate immune responses, metabolic processes, and overall host physiology [15,22,24].

The shift in research emphasis from whole microorganisms to discrete functional components has been driven by substantive methodological advances and the resolution of key technical bottlenecks [25]. Early metagenomic approaches enabled an unbiased survey of the genetic potential of microbial communities; however, they could not distinguish whether detected DNA originated from viable or dead organisms, nor could they directly reflect biological activity in situ [25,26].

Subsequently developed technologies, including metatranscriptomics, metaproteomics, and metabolomics, enabled more direct measurements of microbial community functional outputs through analyses of homogenized samples [27]; however, they still sacrifice the critical spatial context of microbe–host interactions [28]. This limitation has driven the emergence of spatially resolved and single-cell technologies [29,30]. For example, Nejman et al. combined multi-omics analyses with high-resolution imaging techniques, such as multiplex fluorescence in situ hybridization and RNAscope, to reveal the metabolic activity of bacteria in tumors in situ, advancing correlative analyses toward functional validation [28,31]. These technological advances allow for the dissection of microbial component functions at molecular resolution within their native spatial context, providing essential tools for establishing causal links between microbial presence and host phenotypes [29,30].

Therefore, this review proposes that the field of microbiome research is at the forefront of a paradigm shift: from ecological descriptions of “whole microorganisms” to mechanistic analyses of discrete microbial functional components, encompassing metabolites such as short-chain fatty acids and bile acids, cell wall constituents such as polysaccharides and lipopolysaccharides, as well as proteins, nucleic acids, and extracellular vesicles [32]. This transition is not merely a methodological refinement, but a necessary step to overcome the limited mechanistic understanding inherent in traditional whole-microbe studies and to enable precise clinical translation. A component-focused perspective allows the deconstruction of complex microbial communities into discrete signaling units, facilitating an understanding of their actions at defined nodes within host biological networks [33].

Notably, while previous reviews have extensively focused on microbiota-derived metabolites or individual molecular mediators, these frameworks often remain confined to specific classes of molecules or selected signaling pathways [34]. Metabolite-focused studies primarily emphasize small-molecule outputs as downstream functional readouts [35], whereas microbe–host signaling network models focus on receptor-mediated interactions and pathway connectivity [34]. In contrast, the functional component paradigm proposed in this review provides a more integrative and modular framework. It conceptualizes diverse microbial-derived entities—including nucleic acids, proteins, structural components, metabolites, and vesicle-associated cargoes—as functional units defined by their bioactive roles and host targets. This perspective enables the interpretation of complex microbiota effects into cell-type-, tissue-, or condition-specific signaling modules, bridging compositional microbiome data with mechanistic insight [36]. By moving beyond single-category or pathway-centric approaches, this framework emphasizes interconnected mechanisms: hierarchical integration via PRR networks, which coordinate sequential microbial sensing and downstream immune responses [37]; immune-metabolic regulation through the immunometabolic axis, linking metabolite signals to immune cell function [38]; and spatiotemporal specificity of microbial signals, reflecting tissue-, cell type-, and condition-dependent effects [39,40], together providing a generalizable and translationally relevant conceptual foundation for microbiome research.

In this Review, we aim to systematically outline the evolution and prospects of this paradigm shift. We first dissect the key classes of microbial functional components and their modes of action; next, we synthesize a general framework by which the host integrates these signals; finally, we focus on how component-based strategies provide a concrete path for investigating disease mechanisms and advancing precision diagnostics and therapeutics. For example, specific microbial metabolites, such as trimethylamine N-oxide (TMAO) [41,42,43], deoxycholic acid (DCA) [44], and D-lactate [45], as well as microbial DNA features [46], have been reported or are being explored as mechanistically informative biomarkers. In parallel, engineered delivery of beneficial microbial functional components [47] and the development of engineered microbes [48,49] have demonstrated therapeutic potential in preclinical and early clinical studies. Emphasizing functional components thus provides mechanistic insight, guides biomarker discovery, and enables targeted interventions, while highlighting challenges and future directions in translating microbiome research into clinical practice.

## 2. Microbial Functional Components as Operational Units

**Figure 1:**
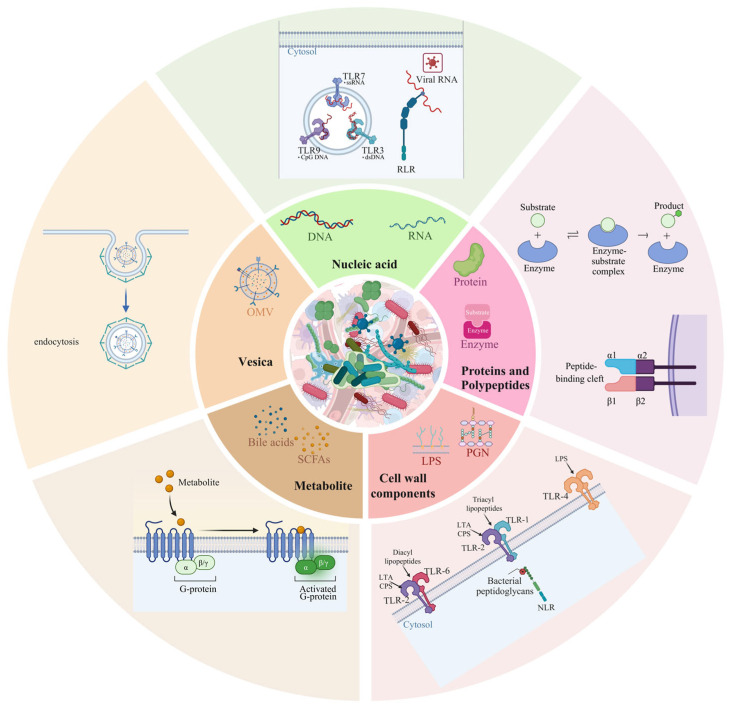
Microbial functional components as operational units in microbe–host interactions. Microbiota-derived functional components act as discrete operational units that mediate microbe–host interactions across multiple biological layers. These functional components encompass diverse molecular classes, including microbial nucleic acids, metabolites, proteins, cell wall constituents, and extracellular vesicles, which collectively engage host sensing systems to regulate immune and metabolic responses.

In studies of microbe–host interactions, deconstructing complex microbial communities into bioactive molecular components with defined functions provides a critical perspective for mechanistically dissecting their effects on the host. These components, ranging from nucleic acids and metabolites to proteins, cell wall-derived molecules, and extracellular vesicles, act as key signaling entities that are sensed by host receptor systems at the cell surface or within the cytoplasm, notably PRRs and metabolite-sensing receptors such as GPCRs, thereby modulating host cellular functions [22,50]. Based on current evidence, this section systematically summarizes several major classes of microbial functional components (Figure 1).

### 2.1. Nucleic Acids: Immune Signaling and Cross-Kingdom Regulatory Mediators

Microbial-derived nucleic acids encompass those from microbiota-associated microorganisms and viruses, including bacterial genomic DNA, plasmid DNA, virus-derived RNA, small RNAs (sRNAs), and nucleic acids encapsulated within extracellular vesicles. These molecules can serve both as canonical ligands for innate immune recognition and, in some contexts, as cross-kingdom regulatory mediators by influencing host gene expression or signaling pathways after interaction with host cells [51,52,53]. The recognition of microbial nucleic acids depends on both their molecular characteristics and subcellular localization [54]. Endosomal receptors primarily detect extracellular or internalized nucleic acids, whereas cytosolic sensors respond to nucleic acids that gain access to the intracellular compartment [54,55,56]. For example, unmethylated CpG motifs enriched in bacterial genomic DNA are recognized by endosomal TLR9, initiating MyD88-dependent NF-κB signaling and inducing pro-inflammatory cytokine production [57]. In addition to host defense, TLR9-mediated sensing contributes to intestinal immune homeostasis and mucosal immune regulation [16]. When bacterial DNA gains access to the host cytoplasm, it can be detected by cGAS, which catalyzes the synthesis of the second messenger cGAMP, subsequently activating the STING-IRF3 pathway and eliciting type I interferon responses critical for defense against intracellular bacterial infections [58]. Similarly, virus-derived double-stranded RNA (dsRNA) is sensed by TLR3 to trigger antiviral immune responses, whereas single-stranded RNA (ssRNA) is primarily recognized by TLR7/8, together constituting a key frontline of antiviral immunity [59,60]. Experimental evidence further demonstrates that RIG-I-like receptors function as cytosolic innate immune sensors that detect non-self-structural features of viral RNA and thereby induce antiviral interferon responses, illustrating that viral RNA can act as a bioactive microbial component rather than merely genetic material [61]. Collectively, microbial nucleic acids function not only as genetic material but also as host-active signaling molecules whose effects are determined by their molecular features and subcellular localization, enabling context-dependent modulation of host immune responses.

### 2.2. Proteins and Peptides: Enzymatic Effectors and Signaling Functions

Functional microbial proteins and peptides comprise a diverse set of components, including secreted enzymes, virulence factors, surface structural proteins, and immunomodulatory peptides. They are primarily derived from the microbiota, but may also include pathogen-derived virulence factors when relevant to microbe–host interactions [62]. These functional components can remodel the local microenvironment through enzymatic activities that alter nutrient availability and metabolite composition, or act directly as signaling effectors (e.g., toxins such as TcdA and TcdB) by engaging host receptors or disrupting intracellular signaling pathways [63,64,65].

Through enzymatic activities, microbial proteins can reshape the local microenvironment by altering nutrient availability and metabolite composition, thereby influencing downstream host-active signaling [66]. Commensal bacteria such as *Bacteroides* species express a wide repertoire of carbohydrate-active enzymes that degrade complex dietary glycans, thereby facilitating downstream fermentation and the production of SCFAs [67]. Similarly, Joyce et al. demonstrated that bacterial Bile Salt Hydrolase activity is a key determinant of host bile acid pool composition and primarily influences host weight gain and lipid metabolism through metabolic remodeling [68].

In the context of immune modulation, microbial proteins can exert diverse effects on host responses, depending on their biological role and mechanism of action. This category extends from commensal-derived immunoregulatory molecules to pathogen-derived virulence factors, which, despite their distinct biological consequences, are similarly able to reprogram host cellular processes through direct molecular interactions [62]. For example, the major toxins TcdA and TcdB from *Clostridioides difficile* bind host cells, enter the cytosol, and glycosylate Rho-family GTPases, thereby disrupting the actin cytoskeleton, weakening epithelial barrier integrity, and promoting inflammatory signaling. Through these effects on host intracellular signaling and mucosal barrier function, they illustrate how pathogen-derived protein factors can shape microbe–host interactions toward inflammatory pathology [69,70,71]. *Lactobacillus plantarum* produces an extracellular “encrypted peptide”, serine–threonine peptide (STp), a bioactive peptide sequence embedded within a larger secreted protein rather than synthesized as a standalone peptide [72,73]. STp was shown to enhance regulatory IL-10 production in human intestinal dendritic cells and to promote an immunoregulatory phenotype in responding T cells, supporting a role for commensal-derived peptides in the regulation of mucosal immune responses [72]. Overall, microbial proteins and peptides shape microbe–host interactions through local biochemical remodeling or direct engagement of host pathways, with context-dependent effects on homeostasis and pathology.

### 2.3. Cell Wall Components: Structural Ligands for PRRs

Functional components derived from bacterial cell walls primarily include peptidoglycans, LPS, teichoic acids, lipoproteins, and surface polysaccharides or capsular polysaccharides. In addition to serving as structural constituents of bacterial cells, these conserved structural motifs function as bioactive microbial signals that are recognized by host pattern-recognition receptors and trigger downstream immune and barrier-regulatory responses. Their biological effects are primarily determined by structure-specific recognition by distinct PRRs, highlighting a central principle of microbe–host interactions: structure-based immune sensing [7,74].

Peptidoglycan (PGN) can be recognized by specific NLR members: NOD1 specifically senses peptidoglycan fragments containing meso-diaminopimelic acid (DAP) (e.g., iE-DAP or GM-triDAP) derived from Gram-negative bacteria and certain Gram-positive bacteria [75]; whereas the minimal immunologically active motif shared by nearly all bacteria—the muramyl dipeptide (MDP) of the cell wall—is detected by NOD2 [76]. Recognition of these PGN-derived motifs by NOD1 and NOD2 triggers RIP2-dependent activation of NF-κB and MAPK signaling, thereby promoting antibacterial responses and contributing to the maintenance of mucosal immune homeostasis [77,78]. Disruption of this sensing and regulatory axis, particularly in the setting of altered NOD2 function, has been implicated in the loss of intestinal immune balance and in susceptibility to chronic intestinal inflammation, including Crohn’s disease [79].

The immunostimulatory activity of LPS primarily depends on its lipid A structure [80]. Pathogenic Gram-negative bacteria typically synthesize hexa-acylated lipid A, which can stably engage the TLR4/MD2 complex and induce receptor dimerization [81], thereby potently activating inflammatory signaling pathways such as NF-κB and IRF3 [82]. In contrast, tetra- or penta-acylated lipid A produced by various gut commensals, including *Bacteroides* species, exhibits markedly reduced TLR4 agonist activity, and some variants even act as antagonists [83]. Rather than abolishing TLR4 recognition, these structural variants are generally associated with weaker or qualitatively distinct signaling outputs, thereby permitting basal microbial sensing while limiting excessive mucosal inflammation [7,84]. In some symbiotic contexts, specific commensal lipid A structures have also been linked to TLR4-dependent regulatory programs that support intestinal immune homeostasis, consistent with a role for lipid A structural variation in shaping microbe–host immune tolerance and maintaining mucosal homeostasis [7].

In Gram-positive bacteria, teichoic acids similarly serve as important structural determinants of immune recognition. Lipoteichoic acid (LTA), anchored to the cytoplasmic membrane through its lipid moiety, serves as an important TLR2 ligand and participates in the early innate recognition of Gram-positive bacteria, thereby shaping downstream inflammatory responses [85]. Wall teichoic acid (WTA), in contrast, is covalently linked to peptidoglycan and helps define bacterial surface properties that influence adhesion and colonization, while its exposed surface structure may also modulate host recognition [86,87]. Beyond their structural roles, both LTA and WTA function as interface molecules that influence how Gram-positive bacteria are sensed by the host and how subsequent host responses are shaped.

Bacterial lipoproteins represent another classic class of TLR2 ligands. Their recognition specificity is determined by the acylation pattern at the N-terminal: triacylated lipoproteins are recognized by the TLR1/TLR2 heterodimer, whereas diacylated lipoproteins are detected by the TLR2/TLR6 heterodimer. This precise structure-receptor correspondence enables the host to finely discriminate between different microbial lipoprotein signals and initiate the appropriate innate immune response [88,89]. Lipoproteins play a critical role in the host response to pathogenic infections [90], while also contributing to the maintenance of basal immune tone in the context of commensal microbes [91].

In addition to the canonical cell wall backbone molecules described above, bacterial surface polysaccharides represent another key class of functional components. Capsular polysaccharides (CPS), in particular, occupy the outermost bacterial interface and can modulate host immune responses [92]. These carbohydrate structures, located at the outermost interface of the bacterial cell, can be sensed by host pattern recognition systems, including TLR2 and C-type lectin receptors, and play key roles in immune modulation [93].

Under conditions of barrier dysfunction or disrupted homeostasis, aberrant translocation of these structural components into the circulation has been associated with systemic low-grade inflammation, as their ectopic exposure can trigger low-level activation of host pattern-recognition pathways and sustain pro-inflammatory signaling [94,95]. Cell wall components act as conserved structural ligands decoded by host PRRs, translating molecular patterns into immune activation or tolerance, highlighting the principle of structure-based host recognition.

### 2.4. Metabolites: Multifaceted Bioactive Small Molecules

Microbially derived metabolites are a diverse group of bioactive small molecules that function as important mediators of microbe–host communication by engaging host immune, metabolic, and epithelial barrier pathways; representative classes include SCFAs, bile acid derivatives, tryptophan metabolites, polyamines, and other signaling metabolites. These metabolic products can influence host physiology through multiple mechanisms, including receptor-ligand interactions, epigenetic regulation, and metabolic reprogramming [15,64,96]. As such, they represent one of the most active and intensively studied classes of mediators facilitating microbe–host communication.

SCFAs, primarily acetate, propionate, and butyrate, function both as energy sources and as key signaling molecules. Butyrate promotes regulatory T cell differentiation by inhibiting histone deacetylase activity [97], while SCFAs also signal through G protein-coupled receptors such as GPR41, GPR43, and GPR109A expressed on epithelial, immune, and enteroendocrine cells, thereby coupling microbial fermentation-derived signals to host responses involved in gut hormone secretion, inflammatory tone, epithelial barrier function, and energy metabolism [98,99].

Bile acids are transformed by the gut microbiota into various secondary derivatives with immunomodulatory activity. For instance, 3-oxo lithocholic acid suppresses Th17 cell differentiation by antagonizing the transcription factor RORγt, whereas isoallolithocholic acid promotes the generation of regulatory T cells [100].

Tryptophan metabolites, particularly indole derivatives such as indole-3-aldehyde, serve as ligands for the aryl hydrocarbon receptor (AhR) and contribute to maintaining intestinal barrier integrity and immune homeostasis, in part through AhR-dependent mucosal signaling [101,102]. Polyamines, such as spermidine, influence barrier function and inflammatory responses through regulation of autophagy [103]. Together with other neuroactive or metabolically active molecules, these metabolites form an interconnected signaling network that influences intestinal barrier integrity, mucosal immune homeostasis, and host metabolic regulation through receptor-mediated signaling, epigenetic modulation, and cellular metabolic processes.

### 2.5. Vesicle-Mediated Delivery Systems of Microbial Components

Vesicle-based functional units primarily include OMVs from Gram-negative bacteria and membrane vesicles from Gram-positive bacteria, which represent specialized delivery systems for microbial signals. These nanoscale vesicles differ from individual microbial functional components in that they package, protect, and deliver multiple cargo molecules (including nucleic acids, proteins, lipids, and cell wall fragments) in an integrated manner, thereby modifying how microbial signals access host cells and elicit downstream responses [104]. Among them, OMVs are prototypical delivery carriers released by Gram-negative bacteria. In addition to virulence factors or commensal modulatory molecules, OMVs can encapsulate DNA, RNA, peptidoglycan, and various lipids and metabolites, thereby enabling the coordinated delivery of diverse microbial signals [104]. This co-delivery mode has profound biological significance. Pathogen-Associated Molecular Patterns embedded in the OMV membrane, such as LPS and lipoproteins, can engage host TLRs and activate innate immune signaling [105]. This process is particularly relevant in mucosal environments and under conditions such as infection, dysbiosis, or epithelial barrier disruption, where vesicle-associated microbial signals are more likely to access host cells and immune sensors [106,107]. In addition, effector molecules contained within OMVs—including sRNAs, proteases, and other functional cargo—are protected by the vesicle structure and can be delivered to host cells, where they further modulate cellular responses [51,104]. For example, in *Pseudomonas aeruginosa*, OMV-mediated delivery of sRNA cargo to airway epithelial cells has been shown to reduce IL-8 production and attenuate neutrophil recruitment [51]. Seminal work by Kaparakis et al. demonstrated that OMV-mediated delivery of peptidoglycan activates the NOD1-RIP2 signaling pathway and induces immune responses [17]. This observation suggests that OMVs facilitate the delivery of microbial ligands to host intracellular sensing pathways, rather than functioning merely as passive carriers. Subsequent studies further showed that OMVs derived from pathogens such as *Vibrio cholerae* can similarly activate NOD-like receptor pathways and promote inflammatory cytokine production [108]. Collectively, vesicles function as composite functional units that integrate multiple microbial signals and enable their coordinated, protected, and spatially targeted delivery. This highlights vesicle-mediated transport as a key mechanism by which microbial functional components achieve spatiotemporal precision in microbe–host interactions.

## 3. Host Integration of Microbial Signals: Common Mechanistic Frameworks

Following the dissection of the specific functions of various microbial functional components, a key question arises: how does the host sense and integrate these diverse molecular signals of distinct origins and properties, and convert them into coordinated physiological outputs? Current evidence indicates that, despite differences in chemical nature and biological effects, interactions between microbial functional components and the host are not entirely discrete; rather, they follow generalizable mechanistic principles [7,109]. These processes primarily involve immune recognition, metabolic regulation, and epithelial barrier-associated signaling pathways. In this section, we synthesize existing studies to delineate the key mechanisms through which the host systematically integrates microbial signals, including pattern recognition networks, immunometabolic crosstalk, the balance between inflammation and immune tolerance, and the spatiotemporal features that shape host responses.

### 3.1. Hierarchical and Redundant Principles of Pattern Recognition Networks

The host primarily senses microbial functional components through the PRR system, which does not function as a simple linear detector but rather as a multilayered, redundant network of receptors [110]. This hierarchy is reflected in the distribution of different PRRs across distinct cellular and subcellular compartments, allowing the host to interpret microbial signals according to where they are encountered and the level of threat they imply. Recognition exhibits complementarity and overlap: a single microbial component can be detected by multiple PRRs—for instance, LPS can activate the membrane-bound TLR4 [111] as well as be sensed by cytosolic caspase-4/5 [112]; conversely, a single PRR can recognize multiple structurally related ligands, such as TLR2 responding to various bacterial lipoproteins [113]. This cross-recognition establishes a robust surveillance system for microbial threats [110]. In addition, PRR signaling outputs are highly context-dependent [114]. Signal strength, duration, and crosstalk with other pathways collectively shape the ultimate immune outcome [115]. For example, highly immunogenic LPS drives strong pro-inflammatory responses, whereas weak agonist LPS from commensal bacteria tends to elicit a more restrained signal and induce negative regulators such as IRAK-M. In turn, this more limited mode of signaling can raise the activation threshold of innate immune responses and promote controlled hyporesponsiveness to persistent commensal-derived stimulation, thereby contributing to immune tolerance [83,84,116]. Moreover, the subcellular localization of PRRs constitutes a critical dimension for threat stratification [114]. Endosomal TLRs (e.g., TLR9) sense nucleic acids that have been captured and compartmentalized by the host, typically triggering controlled immune responses [117]; in contrast, activation of cytosolic sensors (e.g., cGAS) indicates that pathogen- or host-derived nucleic acids have breached physical barriers and entered the cytoplasm, signaling a higher level of threat such as viral replication or severe cellular damage, thereby eliciting a stronger type I interferon response [58]. This “outside-to-inside” localization hierarchy allows the host to assess the urgency of a threat based on its physical location and mount defense responses of appropriate magnitude. PRR networks integrate multiple layers of recognition, redundancy, and context-dependence, highlighting the principle that signal interpretation relies on hierarchical decoding rather than simple ligand-receptor pairing [114].

### 3.2. Bidirectional Regulation Along the Immunometabolic Axis

Microbial functional components function not only as immunostimulatory signals but also as key regulators of host metabolism, thereby contributing to the immunometabolic axis, a bidirectional regulatory framework in which immune signaling and metabolic processes continuously shape one another [118]. Microbial metabolites act as direct signaling molecules shaping immune phenotypes. SCFAs regulate immune cell differentiation and inflammatory thresholds by inhibiting histone deacetylases [119,120] or activating GPCRs [120]. Bile acid derivatives influence barrier integrity and immune homeostasis via receptors such as FXR [121] and TGR5 [122], thereby linking microbial metabolism to host pathways that help maintain intestinal homeostasis, limit mucosal inflammation, and regulate systemic metabolic balance, with relevance to inflammatory bowel disease and metabolic disorders [123,124]. Conversely, immune signals can remodel the metabolic environment. Inflammatory mediators, such as interferon-γ (IFN-γ), can reprogram host metabolic pathways, altering local nutrient availability and redox status. For example, during intestinal inflammation, IFN-γ-associated host responses may promote oxidative and nitrosative processes that reshape the luminal metabolic environment, thereby favoring the expansion of microbial populations adapted to inflammatory conditions, including Enterobacteriaceae [125,126]. This bidirectional feedback loop suggests that microbial signals not only initiate immune responses but are also shaped by immune-driven alterations in the host metabolic environment [118]. Furthermore, energy-sensing pathways such as AMPK and mTOR extensively intersect with immune signaling. Microbial functional components can alter nutrient availability and cellular energy balance in host cells, thereby influencing AMPK/mTOR activity and shaping cytokine production, immune cell differentiation, and inflammatory thresholds [127]. Microbial signals and host metabolism form a bidirectional, feedback-regulated network, illustrating that immune modulation is tightly coupled to metabolic context.

### 3.3. Dynamic Equilibrium Between Inflammation and Immune Tolerance

As an integrated outcome of upstream sensing by PRR networks, modulation via the immunometabolic axis, and the spatiotemporal context in which microbial signals are encountered, the host establishes a dynamic equilibrium between inflammation and immune tolerance, reflecting the net functional state of the system rather than a separate, parallel mechanism [7,15]. Under physiological conditions, this balance is maintained through controlled sensing of commensal-derived cues, which preserves basal host defense while engaging negative-feedback pathways that restrain PRR- and cytokine-driven signaling [128,129]. In this context, commensal-derived functional components promote tolerance programs in part by inducing feedback regulators such as A20 and, in some settings, SOCS family proteins through low-level PRR- and cytokine-dependent signaling, while also promoting Treg differentiation and reinforcing epithelial barrier integrity, thereby limiting excessive inflammatory activation [130,131,132]. Collectively, these mechanisms restrain excessive inflammatory responses [128]. However, once this homeostatic balance is disrupted, sustained inflammatory signaling may contribute to the pathogenesis of chronic inflammatory disorders, including inflammatory bowel disease, in which inappropriate host responses to microbial signals drive persistent mucosal inflammation and tissue injury [133,134]. Notably, certain microbial exposures can induce epigenetic reprogramming in innate immune cells, a phenomenon termed trained immunity [135]. For example, β-glucan and peptidoglycan-derived signals can engage pattern-recognition receptors such as dectin-1 and NOD2, thereby activating signaling programs linked to glycolytic rewiring and to changes in chromatin accessibility and histone modifications at inflammatory gene loci [136,137]. Through these interconnected changes, innate immune response thresholds are durably shifted, helping explain how prior microbial exposure shapes host immune set points [138]. Overall, the balance between inflammatory and tolerogenic outcomes reflects a unifying principle: host responses are emergent properties of integrated sensing and regulatory networks, rather than the consequence of isolated microbial signals.

### 3.4. Spatiotemporal Specificity as a Governing Principle

The systemic effects of microbial functional components exhibit pronounced spatiotemporal dependence, representing a critical determinant of their functional diversity. At the spatial level, signaling outcomes are shaped by anatomical location, tissue compartmentalization, and subcellular localization [130]. For example, microbial metabolites confined to the intestinal lumen predominantly contribute to barrier homeostasis, whereas the same molecules, once translocated into the lamina propria or systemic circulation, may provoke inflammatory responses [139,140]. At the cellular level, the same microbial signal may be decoded differently by epithelial cells, myeloid cells, and lymphocytes owing to differences in receptor repertoire, downstream signaling pathways, and cellular function [139,140,141]. Epithelial cells typically channel these inputs toward barrier-supportive responses, whereas myeloid cells more readily mount inflammatory and antigen-presenting programs [142]. Lymphocytes, in contrast, are influenced mainly through microbially shaped cytokine environments or through direct sensing of selected metabolites, which in turn affects effector and regulatory differentiation [143,144,145]. Temporally, host responsiveness to microbial functional components dynamically varies across early-life developmental stages [146], circadian rhythms [147], and physiological states [148]. Early-life exposures are thought to exert lasting imprinting effects on immune system maturation, while rhythmic metabolic oscillations can further modulate host sensitivity to microbial signals [141,149]. Collectively, spatiotemporal specificity highlights that host responses arise from context-dependent decoding of microbial signals, whereby identical molecular cues can elicit distinct outcomes depending on location, cell type, and timing.

### 3.5. Conceptual Integration: Microbial Functional Components as Modular, Context-Dependent Signaling Units

The host signal integration mechanisms outlined above—including hierarchical PRR networks, bidirectional immunometabolic regulation, spatiotemporal specificity, and the resulting dynamic equilibrium between inflammation and immune tolerance—converge on a unified conceptual framework. In this system, microbial functional components function as modular signaling units, in that different microbial molecules or structures can act as distinct signaling inputs for the host, engaging partly overlapping but non-identical receptors and downstream pathways. Their biological effects therefore do not reside solely in the functional components themselves, but emerge from hierarchical host sensing, integration, and contextual interpretation. Specifically, PRR networks provide the primary sensing input, identifying the identity, abundance, and subcellular localization of microbial signals. The immunometabolic axis modulates signal intensity and cellular responsiveness, while the dynamic equilibrium between inflammation and tolerance represents the integrated functional outcome of these upstream mechanisms. Spatiotemporal specificity further constrains responses, ensuring that identical signals can yield distinct outcomes depending on tissue, cell type, developmental stage, or circadian timing. This duality is illustrated by structurally distinct LPS, including Lipooligosaccharide forms: certain commensal-associated lipid A-containing forms can support tolerogenic or immunoregulatory programs, whereas translocated or strongly immunostimulatory LPS can act as a pathological driver by engaging canonical TLR4-dependent inflammatory signaling [7,150]. By conceptualizing microbial signals as modular and context-dependent units, this framework provides a mechanistic basis for understanding, predicting, and ultimately manipulating microbiota-driven host responses.

## 4. Microbial Component-Driven Signaling and Health Outcomes

Building upon the host signal integration framework outlined above, microbial functional components can be conceptualized as key upstream variables that shape host physiological states. These functional components encompass both structural molecules, such as LPS, peptidoglycan, flagellin, capsular polysaccharides, and OMVs, and microbially derived metabolites, including SCFAs, bile acid derivatives, choline- and carnitine-derived metabolites, and tryptophan metabolites. Distinct microbial functional components, differing in origin and biochemical properties, exert sustained or intermittent influences on immune tone, metabolic networks, and barrier integrity. For example, microbial glycolipids and bile acid metabolites can modulate immune homeostasis [7,15], microbiota-driven metabolic activities can reshape nutrient and lipid metabolism [68,151], and mucus-degrading microbial functions can impair barrier integrity by disrupting the mucus layer and increasing epithelial exposure to luminal microbes [152]. Collectively, these effects influence processes ranging from homeostatic maintenance to chronic inflammation and tumorigenesis. Importantly, accumulating evidence consistently indicates that the biological effects of microbial functional components are not intrinsically beneficial or detrimental; rather, they are highly context-dependent. Outcomes are shaped by multidimensional factors, including exposure dose [153], tissue context [133], host genetic susceptibility [154], and the surrounding microenvironmental state [155].

### 4.1. Tolerance and Nutritional Signals in Homeostatic Maintenance

**Figure 2:**
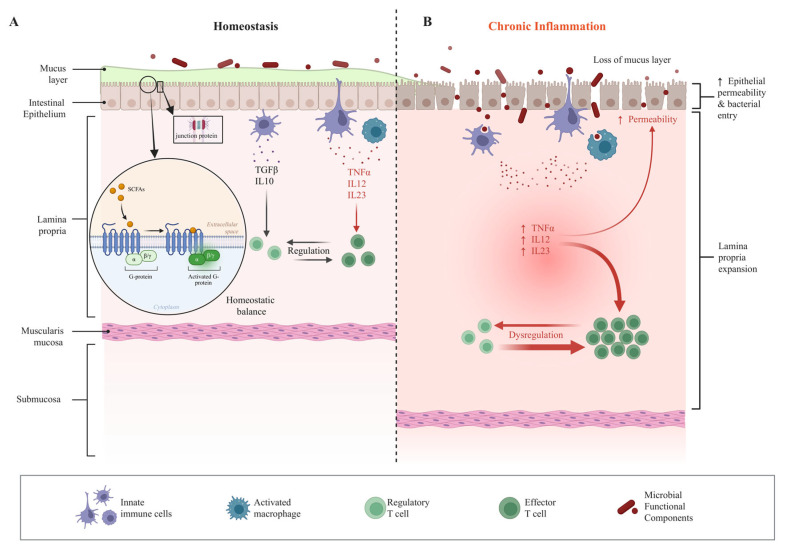
Context-dependent effects of microbial functional components in immune homeostasis and chronic inflammation. (**A**) Under physiological conditions, microbial functional components provide low-intensity signals that promote regulatory immunity and maintain epithelial barrier integrity. (**B**) Barrier disruption or dysbiosis leads to aberrant exposure and persistence of microbial functional components, triggering innate immune signaling and the production of pro-inflammatory mediators.

Under physiological homeostasis, low-intensity signals derived from commensal microbes are critical for maintaining host immune set points [7,156,157] (Figure 2A). These signals include structural components such as capsular polysaccharides and specific forms of LPS, as well as microbial metabolites such as SCFAs. A classic example is *Bacteroides fragilis* capsular polysaccharide A (PSA), which has been shown to induce Foxp3^+^ regulatory T cell (Treg) differentiation and to promote regulatory cytokine production involving IL-10 and, in some settings, TGF-β via TLR2-dependent signaling, thereby contributing to the maintenance of intestinal immune tolerance [150,156]. Concurrently, structural variations in LPS from different bacterial genera—particularly in the lipid A moiety—dictate their TLR4 activation potency, with certain commensal LPS acting as weak agonists or even antagonists, which likely contributes to limiting excessive inflammatory responses [7,83]. Beyond structural molecules, microbial metabolites also play a central role in homeostatic regulation. SCFAs engage GPCRs to enhance epithelial tight junction protein expression and mucin secretion, thereby reinforcing intestinal barrier integrity [158,159,160]. Butyrate additionally inhibits histone deacetylases, promoting Treg differentiation and function to maintain immune tolerance [157]. Collectively, these mechanisms strengthen the intestinal barrier and shape an immune microenvironment conducive to homeostasis, characterized by intact barrier function, low basal inflammation, and a regulatory immune predominance [157,159,161]. Emerging evidence further highlights the role of commensal-derived OMVs in steady-state regulation. By carrying LPS, proteins, and nucleic acids, OMVs can engage host immune pathways at low intensity without bacterial invasion, functioning as “remote immunomodulatory” signaling units [162,163].

### 4.2. Chronic Inflammation Associated with Microbial Functional Components and Aberrant Exposure

When barrier integrity is compromised or microbial community structure is dysregulated, previously sequestered microbial functional components can become significant drivers of chronic inflammation (Figure 2B). The “metabolic endotoxemia” model proposes that increased intestinal permeability allows low-grade, persistent translocation of LPS into the circulation, where it is captured by lipopolysaccharide-binding protein (LBP), transferred to CD14, and presented to the TLR4/MD-2 receptor complex [94,164]. This process activates NF-κB-dependent inflammatory signaling and promotes the production of pro-inflammatory mediators, most prominently TNF-α and, in antigen-presenting cells such as macrophages and dendritic cells, cytokines including IL-12 and IL-23, thereby sustaining systemic low-grade inflammation [94,165]. Consistent with this model, representative studies have shown that high-fat feeding elevates circulating LPS levels and is associated with metabolic inflammation in mice [166]. In humans, increased circulating LBP, a marker of subclinical endotoxemia, has been associated with obesity and related metabolic disorders [167]. Dietary perturbations can further modulate host inflammatory status by reshaping microbial metabolic outputs. For example, high-fat diets can increase DCA production, which has been implicated in DNA damage and pro-inflammatory signaling [168,169]. Likewise, dietary phosphatidylcholine and carnitine metabolism generate trimethylamine (TMA), which is converted in the liver to TMAO and correlates positively with atherosclerotic risk [36,41,43]. At the tissue level, aberrant deposition of bacterial cell wall components is also implicated in sustaining chronic inflammation. Peptidoglycan, for instance, can be detected in joint or vascular tissues and persistently activate innate immune responses via NOD1/2 receptors—a phenomenon particularly prominent in aging and chronic inflammatory disease contexts [170,171,172]. This pattern suggests that the pathological impact of microbial functional components is closely linked to their tissue localization and persistence within the host.

### 4.3. Microbial Functional Components Influencing Tumor Initiation and Therapy

**Figure 3:**
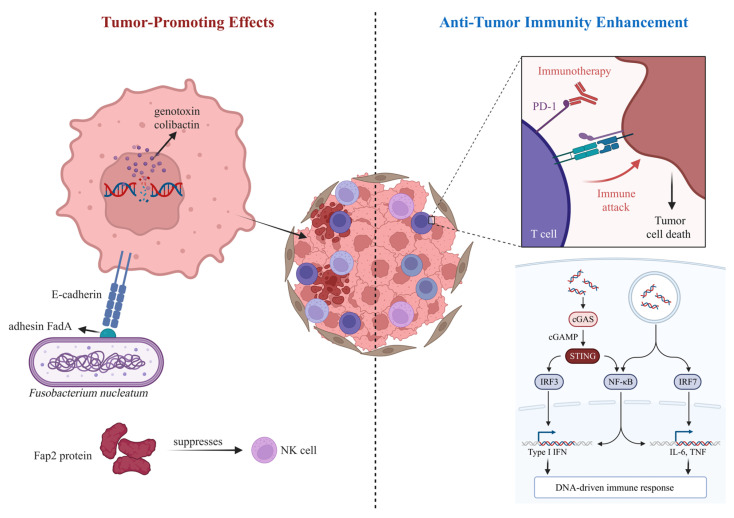
Context-dependent roles of microbial functional components in tumor initiation and cancer therapy. Microbial functional components exert bidirectional effects in tumor biology. They can promote tumor development by inducing DNA damage, activating oncogenic signaling, and suppressing anti-tumor immunity, while also enhancing anti-tumor immune responses through activation of innate immune pathways such as the STING pathway. These effects are highly dependent on the local microenvironment.

Microbial functional components exhibit pronounced bidirectionality and context-dependence in tumor biology (Figure 3). On the tumor-promoting side, *Escherichia coli* strains harboring the *pks* genomic island produce the genotoxin colibactin, which induces host DNA double-strand breaks and generates characteristic mutational signatures; molecular evidence supporting this mechanism has been observed in colorectal cancer tissues [173,174,175,176]. *Fusobacterium nucleatum* can engage host E-cadherin via the adhesin FadA, activating β-catenin signaling and promoting tumor cell proliferation [177], while its Fap2 protein suppresses NK cell activity, contributing to immune evasion [3,177]. Conversely, certain microbial functional components can enhance anti-tumor immunity. In animal models, bifidobacterial components synergize with PD-1/PD-L1 blockade to improve tumor control [178]. Moreover, bacterial DNA or cyclic dinucleotides can activate the STING pathway, triggering type I interferon responses and amplifying anti-tumor immune cascades [179,180,181]. Microbial metabolites within the tumor microenvironment also display context-dependent effects. Butyrate, for example, can enhance tumor immunogenicity via histone deacetylase inhibition [182,183,184]; yet under specific conditions, it may support regulatory immune cell functions [97,119], highlighting that its effects are contingent upon concentration, cell type, and the metabolic milieu [119,183,184].

### 4.4. Microbial Functional Components as Important Modulators of Cancer Immunotherapy Response

Recent evidence indicates that microbial components are important modulators of immune checkpoint inhibitor (ICI) efficacy. Multiple studies have reported enrichment of species such as *Akkermansia muciniphila* and *Bifidobacterium longum* in the gut microbiota of ICI-responding patients [185,186]. Mechanistic investigations further demonstrated that the *A. muciniphila*-derived outer membrane protein Amuc_1100 can enhance dendritic cell maturation and IL-12 secretion via TLR2 signaling, thereby promoting CD8^+^ T cell-mediated antitumor responses [63,187]. Building on these findings, exploratory clinical studies using FMT to reshape the gut microbiota of ICI-refractory patients have shown preliminary signs of therapeutic benefit [188,189]. In addition, specific microbial metabolites and structural components have been proposed as potential biomarkers for predicting immunotherapy response [186,190], although their clinical robustness and reproducibility require validation in larger cohorts.

## 5. Clinical Translation: Intervention Strategies Targeting Microbial Functional Components

**Figure 4:**
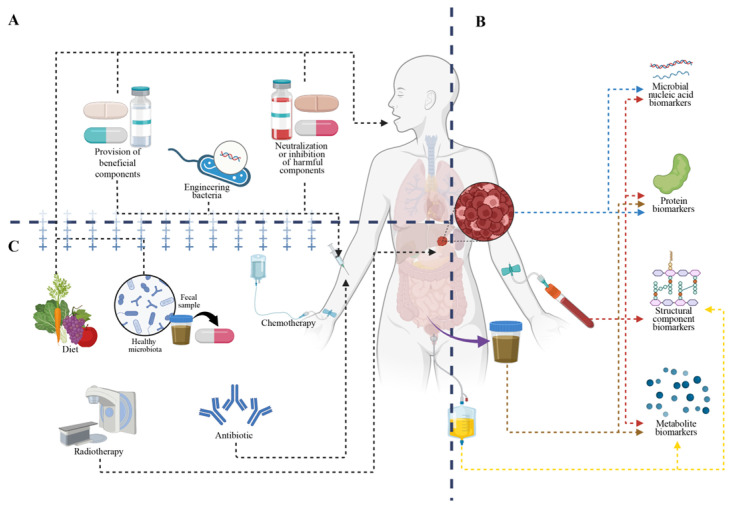
Component-based clinical translation framework. (**A**) Component-based therapeutic strategies: Targeted interventions include provision of beneficial microbial molecules, neutralization of harmful components, and engineered delivery systems for localized therapeutic production. (**B**) Microbial functional components as clinical biomarkers: Microbial nucleic acids, proteins, structural components, and metabolites detected in clinical samples function as mechanistically informative biomarkers. (**C**) Combination therapeutic strategies: Component-based interventions can be integrated with conventional therapies or dietary modulation.

Insights into the functional roles of microbial components are driving a shift from mechanistic understanding toward clinical application [191]. Microbial functional components with greater translational potential mainly include metabolites, surface-associated or structural molecules, and secreted bioactive products, particularly those that can be linked to relatively well-defined host response pathways [7,15]. Compared with interventions based on whole microorganisms, strategies targeting specific microbial functional components offer advantages in mechanistic interpretability, dosage control, and safety assessment [192]. For example, the microbiota-derived metabolite urolithin A can be administered directly as a defined molecular intervention, allowing more consistent exposure than strategies dependent on microbial colonization or stable engraftment [3,193]. Based on current evidence, translational approaches can be broadly categorized into three areas: therapeutic strategies, biomarker development, and combination therapeutic strategies (Figure 4).

### 5.1. Therapeutic Strategies: From “Whole-Microbe Interventions” to Component-Based Therapeutics

The direct development of therapeutics from microbial functional components with defined bioactivity represents a major focus in current translational research. These strategies can be broadly categorized into the provision of beneficial components, neutralization or inhibition of harmful components, and engineered delivery strategies.

Provision of beneficial components aims to harness microbial molecules with immunomodulatory or metabolic regulatory functions as therapeutic agents. For instance, *Bacteroides*-derived capsular PSA has been shown in animal models of inflammatory bowel disease to induce regulatory T cell differentiation and attenuate inflammation, although evidence for its therapeutic application in this context remains largely preclinical [194,195]. SCFAs, particularly butyrate and its prodrugs, are being investigated for the treatment of ulcerative colitis and other disorders associated with impaired intestinal barrier function and mucosal inflammation, based on their ability to enhance epithelial barrier integrity, modulate mucosal immune responses, and suppress inflammatory signaling—mechanisms supported by preclinical and early clinical studies [196,197]. Compared with live biotherapeutics, such component-based approaches may also reduce uncertainties related to colonization efficiency and infection risk [198].

Neutralization or inhibition of harmful components has also achieved notable translational progress. The monoclonal antibody against *Clostridioides difficile* toxin B (bezlotoxumab) has been approved for the prevention of infection recurrence, acting through direct neutralization of toxin activity [199]. In addition, strategies aimed at inhibiting microbial metabolic pathways to reduce the generation of deleterious metabolites have attracted considerable attention. For example, small-molecule inhibitors targeting gut microbial enzymes responsible for TMA production have been shown in animal models to lower circulating TMAO levels and ameliorate atherosclerotic phenotypes [200].

Engineered delivery strategies have rapidly emerged alongside advances in synthetic biology. Genetically engineered probiotics can serve as programmable “component delivery vehicles,” producing cytokines, antigens, or functional metabolites locally within the gut or tumor microenvironment through designed genetic circuits [201]. However, their in vivo stability, immunological safety, and dose controllability require systematic evaluation, and most approaches remain at preclinical or early clinical stages.

### 5.2. Clinical Translation: Microbial Functional Components as Multifunctional Biomarkers

Compared with community composition data that merely indicate “who is present”, microbial functional components directly reflect the functional output of the microbiota and its interactions with the host. As such, they are emerging as mechanistically informative biomarkers that bridge microbial ecology and host clinical phenotypes. Their potential applications span the entire disease management continuum, including risk prediction, adjunctive diagnosis, prognostic assessment, and therapeutic monitoring. According to their chemical properties, microbial component-based biomarkers are classified as shown in Table 1.

Overall, quantitative and compositional changes in microbial-derived functional components in body fluids, feces, and tissues provide a more direct reflection of microbial functional output than species abundance alone. Multidimensional biomarker systems—including nucleic acids, protein antigens, structural components, and metabolites—are driving the development of a “function-guided diagnosis to function-targeted intervention” paradigm, representing a promising direction for the clinical translation of the microbiome.

### 5.3. Combined Therapeutic Strategies

Integrating microbiota-derived component interventions with existing therapies represents a promising approach to enhance efficacy. In the context of chemotherapy and radiotherapy support, prebiotics or SCFAs have shown potential as combination interventions for alleviating intestinal mucosal damage and promoting barrier repair [216]. However, the available evidence in this area still comes predominantly from animal models and other preclinical studies [217]. Although some clinical investigations have been reported, including studies of butyrate and resistant starch in radiation-related intestinal toxicity, the overall number of studies remains limited, and differences in intervention modalities and study endpoints contribute to inconsistent findings [218,219]. Therefore, more high-quality clinical studies are required to further validate their efficacy and applicability [219,220]. In the field of immunotherapy, research has shifted from microbiome associations toward the functional interrogation of microbial functional components. Preclinical models suggest that inosine derived from *Bifidobacterium pseudolongum* may enhance Th1 and effector T cell responses through adenosine A2A receptor signaling on T cells under co-stimulatory conditions, thereby improving tumor responses to immune checkpoint blockade [221]. In parallel, microbial flagellin can activate innate immune signaling pathways such as TLR5, thereby enhancing sensitivity to immune checkpoint inhibitors and promoting immune activation within the tumor microenvironment [48]. Furthermore, “niche remodeling” strategies are gaining attention, whereby pathogenic bacteria are reduced using narrow-spectrum antibiotics [222] or bacteriophages [223], followed by supplementation with beneficial components or functional microbial consortia. Personalized dietary interventions can also indirectly modulate the output of microbial metabolites by altering substrate availability [224].

## 6. Challenges and Future Perspectives

Although a functional component-centric research paradigm has opened more refined avenues for understanding microbe–host interactions and demonstrated encouraging translational potential, several fundamental challenges remain in translating these insights into predictive biological frameworks and reliable clinical interventions. A primary challenge lies in establishing causal relationships within these complex systems. Many associations between microbial functional components and host phenotypes remain largely correlative. Future efforts will require validation of causal chains through large-scale prospective cohort studies, Mendelian randomization analyses, and well-designed human intervention trials. The complexity of this endeavor arises from functional redundancy among microbial functional components and the profound influence of host genetics, immune history, and environmental factors [225,226,227]. Looking ahead, moving the field from association-based understanding toward individualized prediction will require computational modeling that goes beyond high-dimensional correlational analyses. Beyond simply integrating multi-omics data, future efforts will need analytical approaches capable of inferring potential causal relationships, reconstructing complex interaction networks, and capturing system-level dynamics. In this context, causal inference frameworks [228], network-based modeling strategies [229], multiscale dynamic or metabolic models [134,230] and machine learning methods for multi-omics integration [231] deserve particular attention, as they may help illuminate the complex relationships between microbial functional components and host phenotypes from complementary perspectives.

Future breakthroughs will heavily rely on technological innovations that enable high-resolution observation and manipulation of microbial functional components in vivo, in situ, dynamically, and at the single-cell level [232]. For instance, integrating spatial multi-omics with multiplexed in situ imaging holds promise for mapping the distribution of specific microbial functional components within complex tissue microenvironments, identifying the host cell populations they encounter, and linking these spatial patterns to local host responses [232]. Single-cell dual-omics approaches for microbes and host cells may enable simultaneous profiling of gene expression or metabolic activity in individual bacteria together with the molecular phenotypes of interacting host cells, thereby providing single-cell resolution of microbe–host interaction events [233]. In addition, the development of more sensitive and specific biosensors to dynamically monitor microbial metabolites in vivo will further propel mechanistic studies forward [234].

The integration of artificial intelligence with advanced computational modeling is increasingly becoming a key driver for advancing microbe–host interaction research [235]. Faced with high-dimensional, complex datasets linking microbial functional components to host phenotypes, machine learning approaches can identify latent nonlinear association patterns and generate predictive inferences [236,237]. In recent years, algorithms leveraging sequence, structural, and multi-omics features have been applied to predict the potential functions of microbially derived molecules, prioritize candidate microbial metabolites from large-scale datasets, and identify putative host receptors or signaling pathways, thereby generating testable mechanistic hypotheses [238,239,240]. However, most of such predictions remain hypothesis-generating, and only a limited proportion has been directly supported by experimental validation to date [238,240,241]. Looking forward, the combination of multi-scale modeling with individualized data integration holds promise for gradually establishing a digital simulation framework of microbe–host interactions, offering theoretical support for target selection, optimization of delivery strategies, and the design of personalized intervention approaches.

Even when causal links are established, translating these links into a predictive framework is hindered by the opaque nature of microbial communities and a lack of high-resolution tools. Moving from discrete component functions toward a network-based, systems-level understanding of microbe–host interactions requires new theoretical frameworks to capture the collective behavior of complex microbial communities. It is necessary to move beyond linear descriptions of single-component effects and investigate how different microbial functional components within a community interact synergistically, antagonistically, or in cascades to generate emergent properties [242]. Employing controllable synthetic microbial communities as experimental models, coupled with systems biology-based mathematical modeling, offers a route to transform qualitative mechanistic insights into quantitative, predictive functional outputs [243]—an essential goal for the rational design and precise modulation of microbial consortia.

On the path to the clinic, the complexity of the laboratory must contend with the practical constraints of the bedside, presenting a distinct set of bottlenecks in pharmacokinetics, safety, and patient heterogeneity. Therapies based on microbial functional components require comprehensive evaluation of in vivo pharmacokinetics, long-term safety, and optimal delivery strategies. For engineered live microbial platforms, genetic stability, gut colonization efficiency, immunogenicity, and overall biosafety must be rigorously assessed [244]. Identifying stable, reproducible, and readily measurable microbial or metabolite biomarkers for patient stratification and therapy monitoring is a critical step toward personalized medicine. Given the high heterogeneity of disease states and host conditions, integrating microbiome-targeted interventions with existing standard-of-care therapies may offer a more feasible and clinically translatable approach.

## 7. Conclusions

Microbiome research is gradually evolving from an ecological perspective centered on community structure characterization toward a research paradigm that emphasizes molecular functional mechanisms. Deconstructing complex microbial communities into functional components with defined bioactivity—such as nucleic acids, metabolites, and proteins—provides a critical path for establishing operational causal units. Specifically, these are discrete, measurable, and experimentally tractable microbial-derived functional components that can be causally linked to host phenotypes and thus lay a conceptual foundation for redefining microbe–host interactions. These components, acting as direct functional effectors, are integrated by conserved host pattern recognition receptors and metabolic sensing systems, forming key signaling networks that regulate immune and metabolic homeostasis. This integration involves a hierarchical recognition of microbial signals by PRRs, their subsequent modulation through immunometabolic pathways, and the maintenance of a dynamic balance between inflammatory activation and immune tolerance. Compared with existing frameworks that predominantly focus on specific categories of microbial products, such as metabolite-centered studies or linear microbe–host signaling pathways, the functional component-oriented paradigm offers a more integrative and modular framework. It brings diverse microbial-derived entities into a common context-dependent framework, enabling systematic comparison and mechanistic dissection across different classes of microbial effectors. Compared with strategies using intact microbes as intervention units, a component-oriented research paradigm offers potential advantages in mechanistic interpretability, dosage controllability, and safety assessment, thereby reducing therapeutic heterogeneity and improving predictability. Systematic analysis of functional components may thus provide an important conceptual foundation for precision microbiome medicine. In practice, this approach could be implemented through the identification of component-based biomarkers for disease diagnosis and patient stratification, the development of defined microbial-derived molecules as therapeutic agents, and the integration of these components into predictive models to guide personalized intervention strategies. This framework not only provides mechanistic insights into microbiome roles in health and disease but also catalyzes new translational avenues, including component-based drug development, diagnostic biomarker construction, and combination strategies with existing therapies. Achieving this potential, however, remains challenged by functional redundancy, spatiotemporal dynamics, and causal validation. Future progress will depend on the integration of spatial multi-omics, single-cell technologies, artificial intelligence, and synthetic biology, providing technical support for building quantifiable and predictive models of microbe–host component interactions. Among these, multi-omics and AI currently hold the greatest translational potential due to their applicability in biomarker discovery and clinical prediction, while synthetic biology represents a promising but longer-term strategy. From “who is there” in community surveys to “what they do” in mechanistic dissection, and finally to “how to intervene rationally” in strategy design, the deepening of this research trajectory may reshape our understanding and practice of microbiome medicine.

## Figures and Tables

**Table 1:** Microbial-derived biomarkers and their clinical applications across sample types.

Biomarker Type	Measured Biomarker (Examples)	Sample	Major Clinical Applications	Stage of Clinical Application
Metabolites	TMAO	Blood	Prediction of cardiovascular event risk [36,41,43]	Clinically validated (limited adoption)
	DCA, butyrate levels	Serum	Early detection of colorectal cancer and precancerous lesions [44]	Preclinical/early clinical investigation
	D-lactate	Synovial fluid/blood	Adjunctive diagnosis of septic and periprosthetic joint infections [45]	Routine clinical use
Nucleic acids	Hepatitis B virus DNA (HBV DNA)	Serum	Diagnosis, treatment monitoring, and prognosis of chronic hepatitis B [202]	Routine clinical use
	Hepatitis C virus RNA (HCV RNA)	Serum	Confirmatory diagnosis, therapeutic monitoring, and cure assessment in hepatitis C [203]	Routine clinical use
	HPV DNA	Cervical secretions	Primary cervical cancer screening (USPSTF grade A) [204]	Routine clinical use
	High-risk HPV DNA	Cervical secretions/tissue	Monitoring recurrence risk after cervical radiotherapy [205]	Clinically validated (limited adoption)
	HIV-1 RNA	Plasma	Early diagnosis/confirmation of HIV infection, infant diagnosis [206]	Routine clinical use
	DNA of specific bacterial species (e.g., *Fusobacterium nucleatum*)	Feces	Early detection and risk stratification of colorectal cancer [207]	Preclinical/early clinical investigation
	Specific gut microbial features (e.g., *Akkermansia muciniphila* enrichment)	Feces	Prediction of response to PD-1/PD-L1 immune checkpoint inhibitors [208]	Preclinical/early clinical investigation
Proteins/Antibodies	*Helicobacter pylori* antigen or urease activity	Feces/breath	Diagnosis of active *H. pylori* infection and post-eradication follow-up [209,210]	Routine clinical use
	Hepatitis B surface antigen (HBsAg)	Serum	Diagnosis, staging, and therapeutic monitoring of chronic HBV infection [202]	Routine clinical use
	HIV-1 p24 antigen + HIV-1/2 antibodies (4th generation assay)	Serum/plasma	Routine HIV screening [206,211]	Routine clinical use
	Glutamate dehydrogenase (GDH) + Toxin A/B	Feces	Diagnosis and differentiation of *Clostridioides difficile* infection [212]	Routine clinical use
Cell wall components	Lipoarabinomannan (LAM)	Urine	Adjunctive diagnosis of active tuberculosis, especially in HIV-positive patients [213,214,215]	Clinically validated (limited adoption)
	LPS/Lipopolysaccharide-binding protein (LBP)	Blood	Assessment of systemic low-grade inflammation and mechanistic studies of metabolic diseases [94]	Preclinical/early clinical investigation

## Data Availability

No new data were created or analyzed in this study.

## Abbreviations

The following abbreviations are used in this manuscript:

AhR	Aryl Hydrocarbon Receptor
CPS	Capsular Polysaccharides
DAP	Diaminopimelic Acid
DCA	Deoxycholic acid
dsRNA	Double-Stranded RNA
FMT	Fecal Microbiota Transplantation
GDH	Glutamate dehydrogenase
GPCRs	G Protein-Coupled Receptors
HBV DNA	Hepatitis B virus DNA
HBsAg	Hepatitis B surface antigen
HCV RNA	Hepatitis C virus RNA
ICI	immune checkpoint inhibitor
IFN-γ	Interferon-gamma
LAM	Lipoarabinomannan
LBP	Lipopolysaccharide-binding protein
LPS	Lipopolysaccharide
LTA	Lipoteichoic Acid
MAMPs	Microbe-Associated Molecular Patterns
MDP	Muramyl Dipeptide
OMVs	Outer Membrane Vesicles
PGN	Peptidoglycan
PRRs	Pattern Recognition Receptors
SCFAs	Short-Chain Fatty Acids
sRNAs	small RNAs
STp	serine–threonine peptide
ssRNA	Single-Stranded RNA
TMA	trimethylamine
TMAO	Trimethylamine N-oxide
Treg	Regulatory T Cell
WTA	Wall Teichoic Acid
